# Chronic high-fat diet-induced obesity decreased survival and increased hypertrophy of rats with experimental eccentric hypertrophy from chronic aortic regurgitation

**DOI:** 10.1186/1471-2261-14-123

**Published:** 2014-09-24

**Authors:** Wahiba Dhahri, Marie-Claude Drolet, Elise Roussel, Jacques Couet, Marie Arsenault

**Affiliations:** Groupe de Recherche en Valvulopathies, Centre de Recherche, Institut universitaire de cardiologie et de pneumologie de Québec, 2725, Chemin Sainte-Foy, Quebec City, Quebec G1V 4G5 Canada

## Abstract

**Background:**

The composition of a diet can influence myocardial metabolism and development of left ventricular hypertrophy (LVH). The impact of a high-fat diet in chronic left ventricular volume overload (VO) causing eccentric LVH is unknown. This study examined the effects of chronic ingestion of a high-fat diet in rats with chronic VO caused by severe aortic valve regurgitation (AR) on LVH, function and on myocardial energetics and survival.

**Methods:**

Male Wistar rats were divided in four groups: Shams on control or high-fat (HF) diet (15 rats/group) and AR rats fed with the same diets (ARC (n = 56) and ARHF (n = 32)). HF diet was started one week before AR induction and the protocol was stopped 30 weeks later.

**Results:**

As expected, AR caused significant LV dilation and hypertrophy and this was exacerbated in the ARHF group. Moreover, survival in the ARHF group was significantly decreased compared the ARC group. Although the sham animals on HF also developed significant obesity compared to those on control diet, this was not associated with heart hypertrophy. The HF diet in AR rats partially countered the expected shift in myocardial energy substrate preference usually observed in heart hypertrophy (from fatty acids towards glucose). Systolic function was decreased in AR rats but HF diet had no impact on this parameter. The response to HF diet of different fatty acid oxidation markers as well as the increase in glucose transporter-4 translocation to the plasma membrane compared to ARC was blunted in AR animals compared to those on control diet.

**Conclusions:**

HF diet for 30 weeks decreased survival of AR rats and worsened eccentric hypertrophy without affecting systolic function. The expected adaptation of myocardial energetics to volume-overload left ventricle hypertrophy in AR animals seemed to be impaired by the high-fat diet suggesting less metabolic flexibility.

**Electronic supplementary material:**

The online version of this article (doi:10.1186/1471-2261-14-123) contains supplementary material, which is available to authorized users.

## Background

Bad dietary habits have been linked to the obesity epidemic in industrialized countries. High fat (HF) diets were initially incriminated for this epidemic but this hypothesis is now being challenged. The direct cardiac toxicity of dietary sugar overabundance has been documented in animal models [1–3] but the effects of HF diets on the heart are still controversial [4]. This is probably due to the variability of the HF diets that have been studied in rodent models of heart diseases. For example, a “Western” diet (rich in both fat and carbohydrates) has been shown to cause contractile dysfunction [5] in Wistar rats while a high fat/low carbohydrate diet seemed less toxic for the heart even if its content in saturated fatty acids was high [6].

HF diets have mostly been shown to be either neutral or somewhat beneficial in rodents with LV pressure overload [7, 8]. A neutral response to a HF diet on cardiac remodeling was also observed in a rat model of ischemic heart failure [9]. The impact of a HF diet on dilated cardiomyopathy and eccentric LVH resulting from chronic LV volume overload (VO) has never been studied.

Chronic LV-VO causes severe dilatation and eccentric hypertrophy. It is encountered mostly in patients suffering from heart valve regurgitation (mitral or aortic). Untreated regurgitation will result in severe LV dilatation and hypertrophy as well as slowly progressive systolic dysfunction and heart failure [10]. The obesity epidemics and the western diets have reached several developing countries worldwide [11]. It is thus important to determine if dietary habits can influence the evolution of LV VO diseases. This study was therefore designed to assess the impact of a chronic high saturated fat diet on the development of eccentric LV hypertrophy, function and survival in rats with severe and chronic LV VO from aortic valve regurgitation (AR).

## Methods

### Animals

Adult male Wistar rats were purchased from Charles River (Saint-Constant QC, Canada) and divided in 4 groups as follows: 1) Sham-operated animals on control diet (SC; n = 15); 2) AR control diet (ARC; n = 56), 3) Sham on High-Fat diet (Adjusted calories diet (60% calories from fat), Cat. No. TD.06414 Harlan Teklad Madison WI), (SHF; n = 12) and AR on High-Fat diet (ARHF n = 32). The animals were maintained either on the control diet (Purina Rat Chow #5012) containing 13% calories from fat, 27% from protein and 60% from carbohydrate (39.5 g/kg from starch; 4.1kCal/g) or the high-fat diet containing 60% calories from fat, 19% protein and 21% carbohydrate (34.3 g/kg from fat; 5.4 kCal/g). The fatty acid profile (% of total fat) was 37% saturated (control diet: 19%), 47% monounsaturated (control diet: 20%) and 16% polyunsaturated (control diet: 61%). The high-fat diet was started one week before surgery in both SHF and ARHF groups and continued for 30 weeks until sacrifice. A second protocol with similar groups of animals was also investigated but for a shorter period of 8 weeks (n = 10/gr.). The protocol was approved by the Université Laval’s Animal Protection Committee and followed the recommendations of the Canadian Council on Laboratory Animal Care.

### Aortic regurgitation

Severe AR was induced by retrograde puncture of the aortic valve leaflets as previously described [12]. A complete echocardiographic exam with a HD11XE echograph (Philips Medical Imaging, Andover, MA) was performed two weeks after AR induction and the day before sacrifice 30 weeks later. At the end of the protocol, hearts were quickly dissected and all cardiac chambers were weighed. LV was snap-frozen in liquid nitrogen and kept at −80°C for further analysis. Serum samples were kept for evaluation of lipids, glucose, insulin and adiponectin using commercial kits.

### Analysis of mRNA accumulation by quantitative RT-PCR

The analysis of LV mRNA levels by quantitative RT-PCR has been described in details elsewhere [13].

### Enzyme activity determinations

Left ventricle samples were assayed for maximal (*V*_max_) enzyme activities. Small pieces of LV (20-30 mg) were homogenized in a glass-glass homogenizer with 9 or 39 volumes of ice-cold extracting medium pH7.4 (250 mM sucrose, 10 mM Tris–HCl, 1 mM EGTA) depending on the enzyme activity assayed. Enzymatic activities were determined as previously described [14, 15].

### Immunoblotting

Crude LV homogenates were separated by SDS-PAGE. Preparation of membrane, nuclear and cytosolic fractions for the analysis of Glut4 and IRS1 translocation or AMPK subcellular localization were performed using protocols described in the following articles [16, 17]. Immunoblotting was performed as described elsewhere [18]. All primary antibodies were used at a 1:1000 dilution and were purchased from Cell Signaling Technology (Beverly, MA) or from Santa Cruz Biotechnology (Santa Cruz,CA).

### Statistical analysis

Results are presented as mean ± SEM unless specified otherwise. Normality of the data was assessed by the Pearson test. Inter-group comparisons were done using two-way ANOVA and using Bonferroni post-test if necessary. Statistical significance was set at a *p* < 0.05. Data and statistical analysis were performed using Graph Pad Prism version 6.01 for Windows, (San Diego CA).

## Results

### Clinical data and animal characteristics

**Figure 1:**
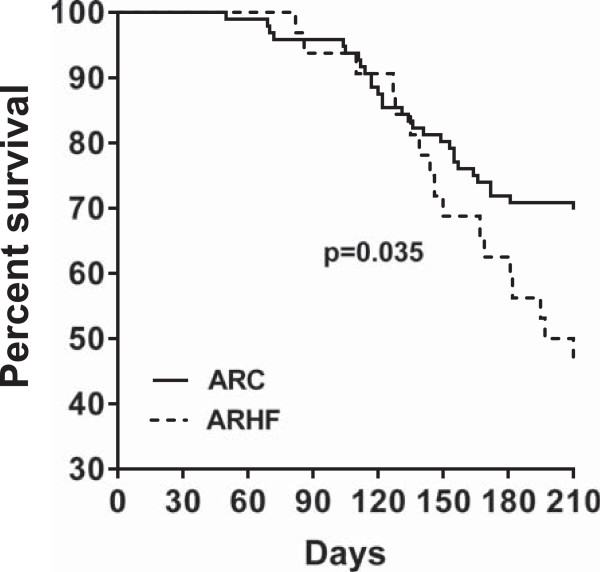
**Survival Kaplan-Meier curves of rats with severe chronic aortic valve regurgitation.** AR rats were fed control chow Untreated (ARC: solid line) or the high fat diet (ARHF: dotted line) for 7 months.

**Figure 2:**
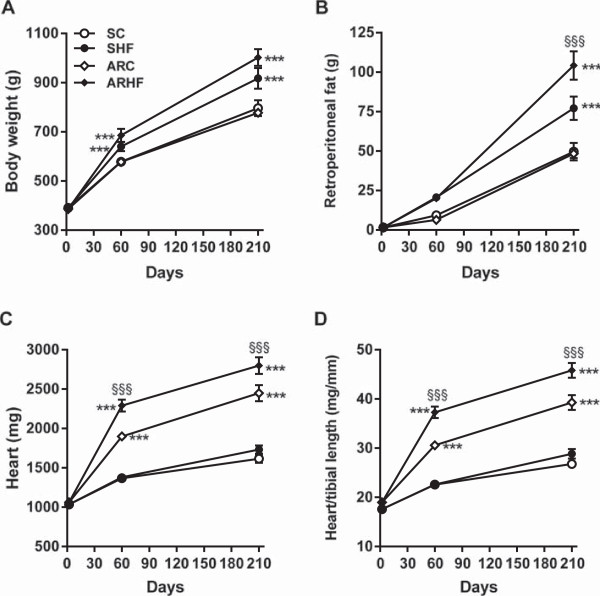
**Heart hypertrophy is worsened by feeding AR rats with high-fat (HF) diet.** Evolution of the body weight **(A)** and retroperitoneal fat deposit weight **(B)** was evaluated at 2 and 7 months. Heart weight **(C)** and indexed heart weight (**D**; for tibial length). Results are reported as mean ± SEM. Two-way ANOVA analyses. ***: p < 0.001 versus sham-operated animals. If interaction between AR and diet was found to have a P value below 0.05, a Bonferroni post-test was conducted: §§§: p < 0.001 vs. control diet corresponding group.

All animals were alive at the end of the 8-week protocol with the exception of two deaths in the ARHF group. In the longer 30-week protocol all control animals were alive at the end. Seventy percent of AR animals fed with the control chow were alive after 7 months compared to less than 50% in the one receiving the high-fat diet (Figure 1). Animals fed a high-fat diet (HF) were significantly heavier (Figure 2A). The retro-peritoneal fat content was markedly increased in the HF groups and even more so in AR animals (Figure 2B). Caloric intake by the animals was also higher in the HF groups (Table 1). Glucose levels were slightly lower in HF animals. Insulin levels remained unchanged with the diet but were slightly more elevated in AR animals. Leptin levels were strongly increased in HF groups. The disease status (AR or not) had no impact on these parameters except for adiponectin levels which were lower in ARC animals compared to ARHF ones.

**Table 1:** **Biometric and echocardiographic parameters**

	Sham Ctrl	Sham HF	AR Ctrl	AR HF
Tibia, mm	60.3 ± 0.64	61.4 ± 0.31	61.0 ± 0.43	61.0 ± 0.56
LV, mg	1163 ± 34.0	1225 ± 34.2	1823 ± 77.0a	2046 ± 69.9a, c
EDD, mm	8.4 ± 0.14	8.5 ± 0.17	11.4 ± 0.18a	11.6 ± 0.18a
ESD, mm	3.8 ± 0.22	3.8 ± 0.20	6.7 ± 0.17a	6.6 ± 0.22a
EF,%	78.6 ± 1.81	80.1 ± 1.52	64.8 ± 1.05a	67.9 ± 1.63a
RWT	0.30 ± 0.005	0.29 ± 0.005	0.23 ± 0.005a	0.21 ± 0.005a
kCal/day	126 ± 4.0	130 ± 4.0	126 ± 2.3	134 ± 4.1
TG, mmol/l	1.1 ± 0.12	1.1 ± 0.12	1.4 ± 0.14	1.0 ± 0.11
Chol., mmol/l	3.1 ± 0.46	2.9 ± 0.12	2.6 ± 0.13	3.1 ± 0.17
Gluc., mmol/l	17.4 ± 1.53	17.3 ± 1.72	15.9 ± 1.19	15.0 ± 1.05
Insulin, μg/l	1.4 ± 0.25	1.1 ± 0.19	1.6 ± 0.21a	2.4 ± 0.70a
Leptin, mg/l	1.1 ± 0.17	2.0 ± 0.22b	0.7 ± 0.13	2.1 ± 0.22b
Adiponectin, mg/l	10.4 ± 0.76	10.4 ± 0.69	7.7 ± 0.34	10.4 ± 0.76c

Echo measurements obtained under 1.5% isoflurane anesthesia. *LV* left ventricle, *EDD* end-diastolic diameter, *ESD* end-systolic diameter, *EF* ejection fraction and *RWT* relative wall thickness. Animal daily food intake (kCal/day) and plasma levels of glucose, triglycerides, insulin, leptin and adiponectin plasma levels were estimated in animals sacrificed at 60 days post-AR. a: p < 0.001 versus sham groups, b: p < 0.01 vs. control diet groups and c: p < 0.01 vs. corresponding AR group by Bonferroni post-test. HF: high-fat diet.

Heart weight was increased in rats with AR (Figure 2C). AR animals on the HF diet had a significantly higher heart weight than those on the control diet. Sham-operated animals fed with the HF diet had a similar heart weight to SC animals. When indexed for tibial length (Figure 2D), this difference in heart weight between ARC and ARHF animals was still significant. LV weights followed the same variations as for total heart weight (Table 1). Ejection fractions in the two AR groups were similarly decreased. Relative wall thickness (an index of LV remodeling) was similarly decreased in AR rats fed or not with the HF diet (Table 1).

### LV hypertrophy markers

**Figure 3:**
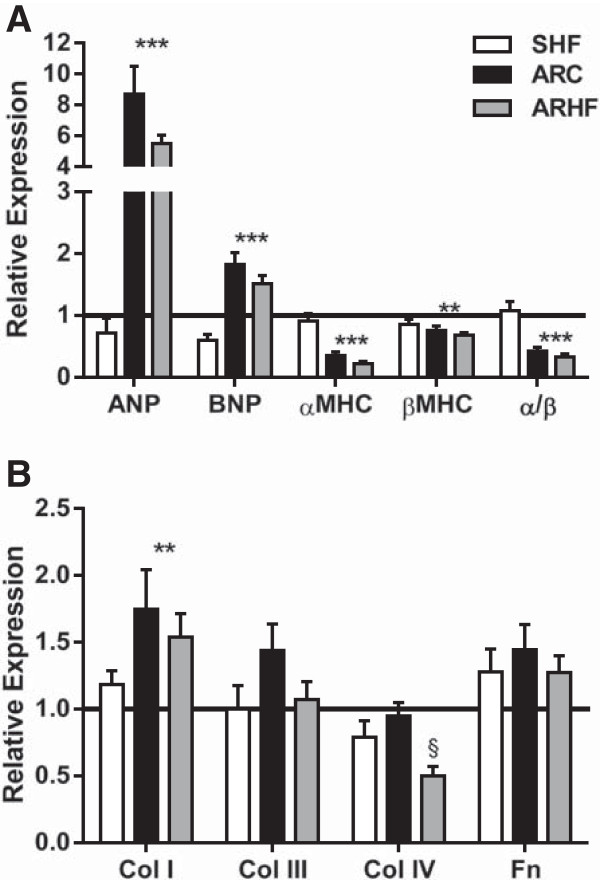
**Evaluation by real-time quantitative RT-PCR of the LV mRNA levels of genes related to LV hypertrophy (A) and extracellular matrix remodeling (B).** **: p < 0.01 and ***: p < 0.001 between sham and AR groups. §: p < 0.05 vs. corresponding group on control diet. Sham (sham-operated animals) group mRNA levels were normalized to 1 and are represented as a black line on the different graphs. ANP, atrial natriuretic peptide; BNP, brain natriuretic peptide; αMHC, myosin heavy chain alpha; βMHC, myosin heavy chain beta; α/β: ratio of the two MCH forms; Col1: collagen I; Col III: collagen III; Col IV: collagen IV Fn: fibronectin.

As illustrated in Figure 3A, ANP and BNP gene expression were clearly increased in the LV of AR animals but the HF diet had no effect on these parameters. Myosin heavy chain subunits gene expression was also modulated similarly in both AR groups.

**Figure 4:**
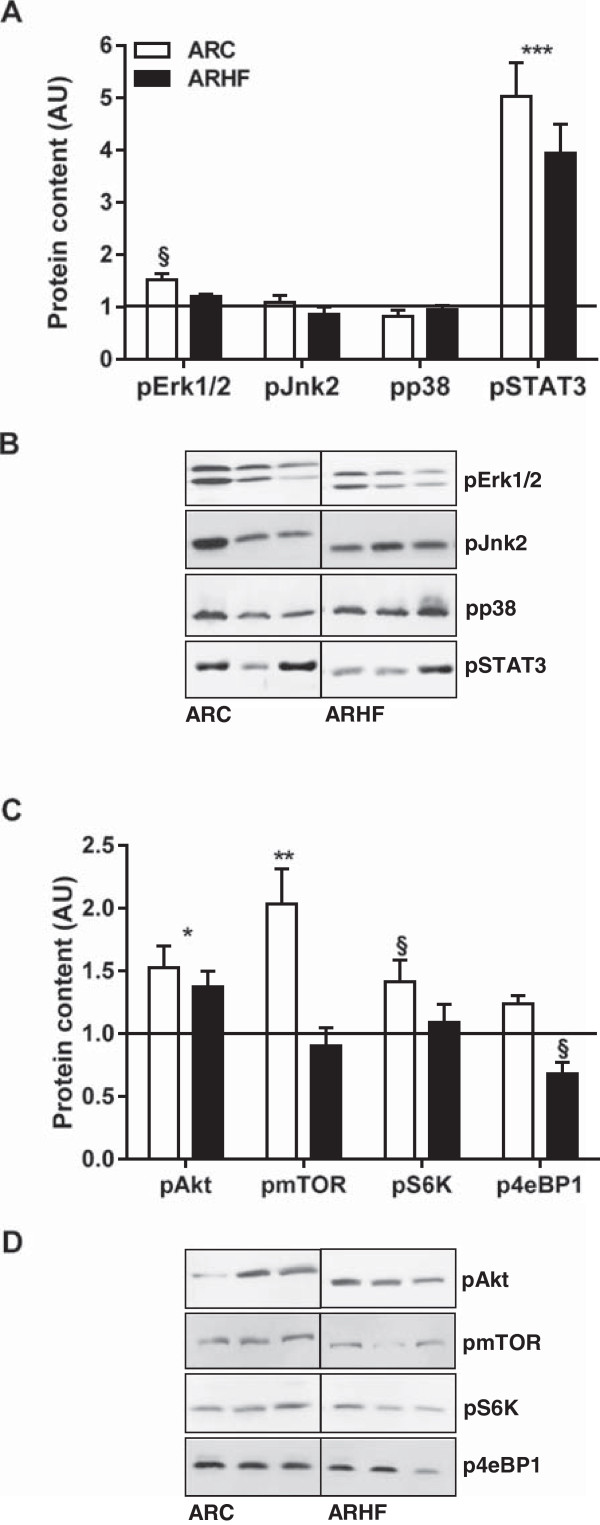
**The high fat diet impact on LV signaling in AR rats.** Phosphorylated protein content of various signaling molecules implicated in cardiac growth and hypertrophy were evaluated by immunoblotting. SC protein levels were normalized to 1 and are represented as a black line on the different graphs **(A and C)**. *: p < 0.05, **: p < 0.01 and ***: p < 0.001 between sham and AR groups. §: p < 0.05 vs. corresponding group on control diet. ARC animals are represented by the white columns and the ARHF the solid ones. Representative blots are also illustrated **(B and D)**.

AR mildly stimulated Erk1/2 phosphorylation but did not affect the activation p38 or Jnk (Figure 4A). The HF diet reversed the AR effect on pErk1/2 (Figure 4A-B). Stat3 phosphorylation was strongly increased in AR rats but the high-fat diet did not influence this parameter. Phosphorylation of Akt, mTOR and S6 kinase were significantly increased in the LV of ARC animals whereas only a trend was observed for 4eBP1 (Figure 4C). The HF diet on the other hand reversed this effect on mTOR and the S6 kinase and reduced basal phosphorylation levels of 4eBP1 (Figure 4C-D). Myocardial interstitial and peri-vascular fibrosis was significantly increased in AR rat but the HF diet was not associated with additional fibrosis (Additional file 1: Figures S1 and S2).

### GSK3β and FoxO inactivation by the HF diet in eccentric LVH

**Figure 5:**
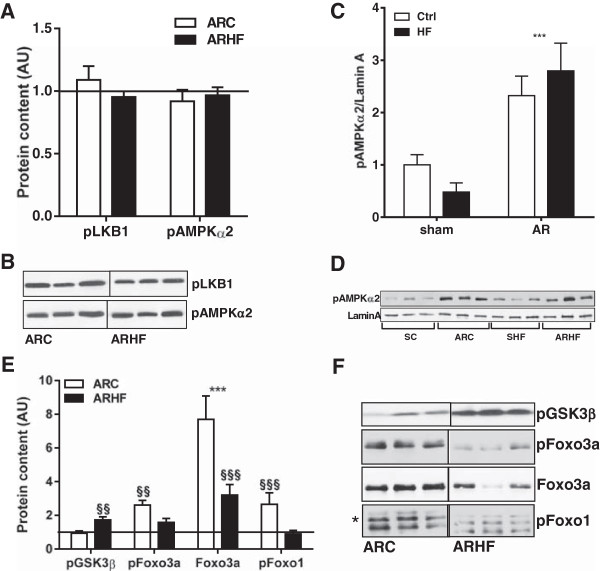
**AMPK phosphorylation and cellular sub-localization, GSK3β and FoxO phosphorylation.** Phosphorylated protein contents of LKB1 and AMPKα2 subunit remains unchanged in the LV myocardium of AR rats fed or not with a high fat diet. SC protein levels were normalized to 1 and are represented as a black line on the different graphs **(A, C and E)**. ***: p < 0.001 between sham and AR groups. §§: p < 0.01 and §§§: p < 0.001 vs. corresponding group on control diet. Representative blots are also illustrated **(B, D and F)**.

Phosphorylation of GSK3β and of FoxO1 and 3a leads to their inactivation by inhibiting the kinase activity of the former and nuclear localization of FoxOs [19, 20]. We observed that AR was associated with higher levels of FoxO 1 and 3a phosphorylation. On the other hand, the HF diet doubled phosphorylation levels of GSK3β while decreasing those of FoxO1 and 3a (Figure 5E-F).

### Cardiac metabolism

**Figure 6:**
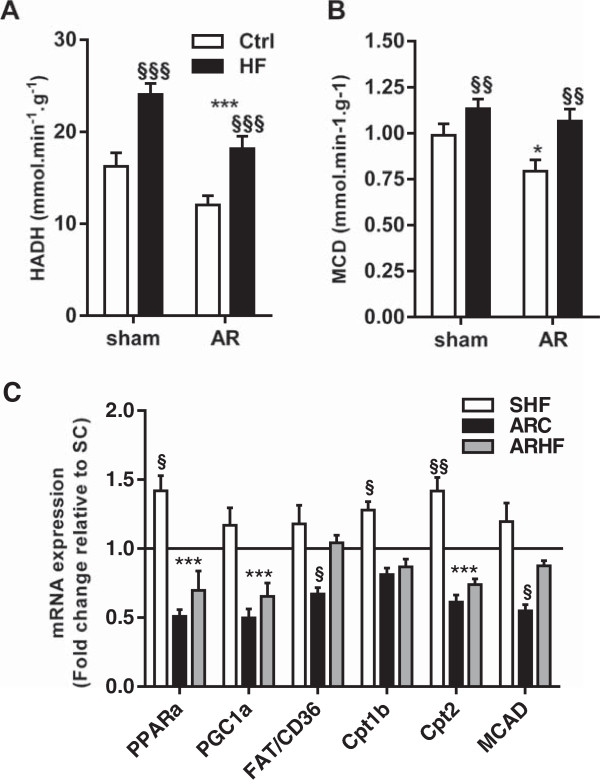
**Impact of a high-fat diet on FAO in chronic eccentric LV hypertrophy.** HADH (**A**; hydroxyacyl-Coenzyme A dehydrogenase) and MCD (**B**; malonyl-CoA decarboxylase) enzymatic activities were measured as described in the Materials and Methods. In panel **C**, evaluation by real-time quantitative RT-PCR of the LV mRNA levels of genes related to FAO. Sham group mRNA levels were normalized to 1 and are represented as a black line on the graph. *: p < 0.05 and ***: p < 0.001 between sham and AR groups. §: p < 0.05, §§: p < 0.01 and §§§: p < 0.001 vs. corresponding sham or AR group.

We have previously showed that eccentric LVH caused by severe AR was associated with a shift in energy substrates preference from fatty acids to glucose [14, 15, 21]. Hydroxyacyl-Coenzyme A dehydrogenase (HADH) was reduced in AR animals (Figure 6A). The HADH enzymatic activity was increased by the HF diet in sham and AR animals, normalizing this parameter in the latter group. The findings were similar for the malonyl-CoA decarboxylase (MCD) enzymatic activity (Figure 6B). We then measured mRNA levels of a set of genes related to FAO in the LV. As illustrated in Figure 6C, the HF diet was associated with an almost overall increase in gene expression of these genes in control animals whereas AR rats on the control diet displayed decreased levels. The administration of the HF diet to the AR animals failed to normalize the gene expression of all but one of these genes (Fat/CD36).

**Figure 7:**
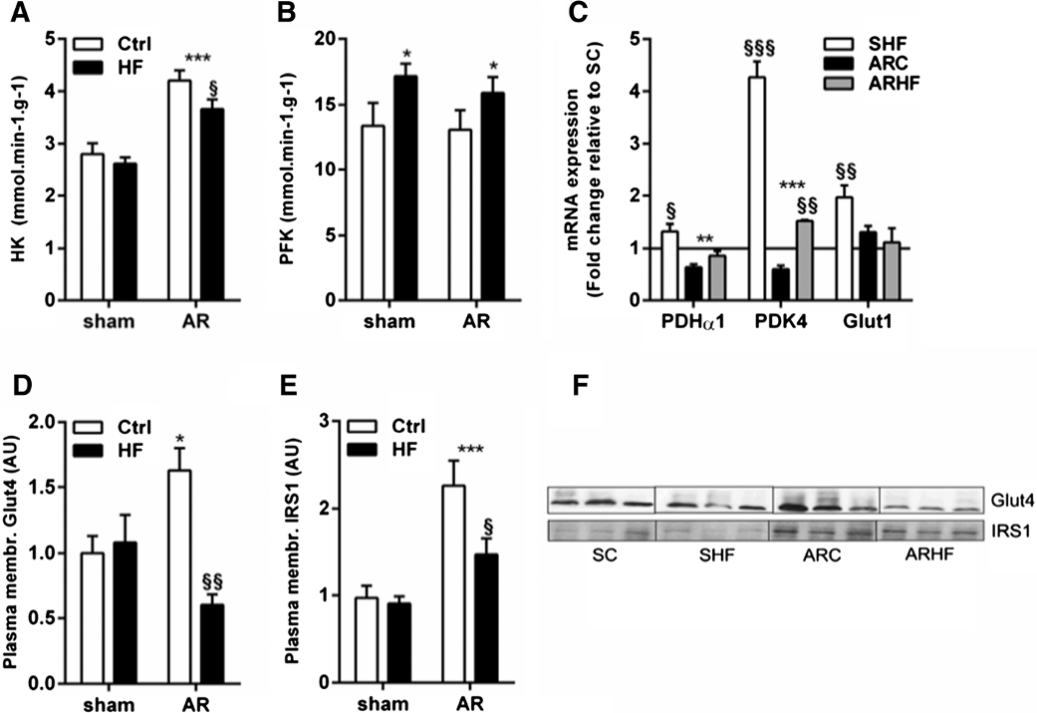
**The high fat diet in AR rats seems to impair the switch from fatty acid to glucose as preferred myocardium energy substrate.** HK (**A**; hexokinase) and PFK (**B**; phosphofructokinase) enzymatic activities were measured as described in the Materials and Methods. Evaluation by real-time quantitative RT-PCR of the LV mRNA levels of other genes related to glucose oxidation is illustrated in panel **C**. **D**, Increased Glut4 translocation to the plasma membrane is reversed by the high fat diet in AR rats as evaluated by immunoblotting. **E**, Recruitment of IRS1 to the membrane in the LVs of AR rats is partly reversed by the high fat diet. **F**, Representative blots are illustrated. *: p < 0.05 and ***: p < 0.001 between sham and AR groups. §: p < 0.05, §§: p < 0.01 and §§§: p < 0.001 vs. corresponding sham or AR group.

Hexokinase (HK) activity is increased in the LV myocardium of AR animals and the HF diet slightly decreased this parameter (Figure 7A). On the other hand, phosphofructokinase activity was increased to a similar extent in HF diet fed animals (Figure 7B). The HF diet was also associated with higher mRNA levels of the pyruvate dehydrogenase α1 (PDHα1), pyruvate dehydrogenase kinase 4 (PDK4) as well as of glucose transporter 1 (Glut1) (Figure 7C). We also evaluated insulin-sensitive glucose transporter-4 (Glut4) translocation to the sarcomeric membrane (Figure 7D and F). AR increased this translocation compared to sham animals fed or not with the HF diet. The HF diet completely reversed this finding in AR and reduced Glut4 translocation to sub-normal levels. We also investigated if this finding could be explained by a variation in the insulin receptor signaling pathway activation by measuring the content of IRS1 recruitment to the plasma membrane (Figure 7E-F). As for Glut4, IRS1 was more present at the membrane in AR rats fed with the control chow. The HF diet decreased this parameter in AR rats.

**Figure 8:**
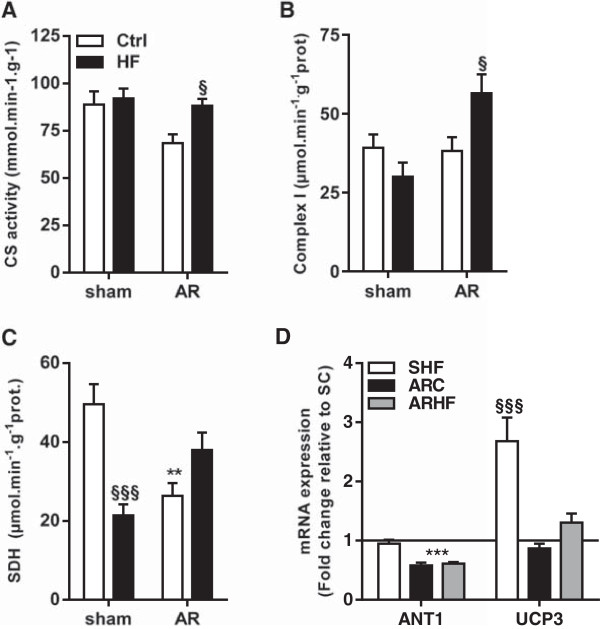
**The high fat diet impact on myocardial mitochondria.** HK (**A**; hexokinase), complex 1 **(B)** and SDH (**C**; succinate dehydrogenase) enzymatic activities were measured as described in the Materials and Methods. Evaluation by real-time quantitative RT-PCR of the LV mRNA levels of ANT1 (adenine nucleotide translocator-1) and UCP-3 (uncoupling protein-3) is illustrated in Panel **D**. **: p < 0.01 and ***: p < 0.001 between sham and AR groups. §: p < 0.05 and §§§: p < 0.001 vs. corresponding sham or AR group.

Citrate synthase activity was reduced in AR animals but HF diet reversed this trend (Figure 8A). The complex 1 activity remained mostly unchanged but was increased in AR animals fed with the HF diet (Figure 8B). Succinate dehydrogenase activity was decreased in SHF and ARC animals but not in ARHF (Figure 8C). Total CK activity was slightly decreased in AR animals (not shown). Gene expression for the adenine nucleotide translocator-1 (ANT1) was reduced in both AR groups (Figure 8D). Uncoupling protein 3 (UCP3) mRNA levels were strongly increased in sham animals fed the HF diet. This was not case in AR animals where UCP3 expression remained unchanged.

## Discussion

Obesity has been known as an independent risk factor for cardiovascular disease [22] but the contribution of obesity-induced comorbidities such as dyslipidemia, hypertension or type II diabetes has to be taken into account. In humans, however, “uncomplicated” isolated obesity has been shown to be linked with cardiac anomalies too [23, 24]. Dietary habits clearly play a major role in the development of obesity and its associated complications.

Divergent results have been reported in experimental models of diet-induced obesity and heart disease depending on the composition of the abnormal diet. Some studies have suggested that diet-induced obesity does not alter cardiac function [6, 25] while others reported various cardiac systolic and/or diastolic function anomalies [26–29]. Feeding animals with LV pressure overload with a HF diet had neutral effects in some studies [7, 8, 30] but adverse effects in another [31]. A HF diet suggested some benefits in a rat myocardial ischemia model [9, 32].

In our model of LV volume overload from AR with severe eccentric LVH, the HF diet was associated with increased LVH and poorer survival. It could be argued that the increase in heart weight in ARHF could be related only to the increased body mass. However a similar increase in heart weight should have been observed in the overweight sham animals fed the HF diet. Excessive weight gain may have played a role but it clearly was not the main factor to explain the differences. It is possible that a synergy between hemodynamic overload, diet and hypertrophy which are all pro-hypertrophic factors exist but the mechanisms for the increased hypertrophy in AR rats fed with the HF diet remain unclear. We observed in the past that the Erk 1/2 and Stat3 pathways are activated in AR [14, 15, 33]. We did not observe such an activation for the Akt/mTOR pathway in the short protocols (8 weeks) but we did in longer ones (current and [34]). Here, the HF diet returned the activation levels both Erk and mTOR pathways to normal suggesting that they were not responsible for the increased LVH observed in ARHF. Additional LVH may be caused by the inhibition of anti-hypertrophic molecules such as GSK3β and FoxOs. We observed increased phosphorylation of these molecules in AR rats fed with the HF diet, likely leading to their inactivation. GSK3β phosphorylation impairs its inhibition in various pathways linked to hypertrophy or cardiac growth including Wnt and NFAT signaling [35]. FoxO favors transcription of atrogin-1, an ubiquitin ligase that promotes calcineurin degradation and can prevent NFAT activation.

Many myocardial metabolic enzymes were modulated both by the state of severe LV-VO and by the diet. The activity level of HADH was increased in SHF animals but not in SC ones. The expression of the fatty acid transporters (Fat/CD36 and CPTs) were all increased in animals on the HF diet with the exception of Fat/CD36 in the ARHF group. HF diet seems to have a stimulating effect on FAO in sham animals but this effect was somewhat blunted in AR animals. We also measured the activity levels of two enzymes of the citric acid cycle. Citrate synthase levels remained relatively stable among groups but SDH activity was strongly reduced in ARC animals. A similar observation has been made previously in a pressure overload rat model [7]. In our model the HF diet helped restore normal levels of SDH activity.

The discrepancies between the impacts of HF diet in pressure versus volume overload models are interesting and raise some questions. Our results suggest that FAO capacity is not increased in AR animals fed a HF diet. The main source of fat in our HF diet was lard and the diet was started one week before AR induction. Unlike mice in Raher et al. [31], our rats did not seem to develop any clear form of metabolic syndrome or overt diabetes despite an important increase in body mass. Our results are nevertheless in line with some of the observations made in their mouse pressure-overload model. We observed more LV hypertrophy in rats on the HF diet. We also found an increase in circulating leptin in our animals. Leptin has been shown to promote the hypertrophy of cardiac myocytes [36]. A severe pro-hypertrophic stimulus suddenly imposed to a heart with preexisting elevated leptin levels could induce an excessive hypertrophic response. Therefore, the response to this pro-hypertrophic stress may have been amplified from the very start since the HF diet had been started before AR. The heart goes through a hyper-contractile phase to compensate for the acute volume overload in the first days following AR induction [12]. The heart has to maintain its contractile capacity and to simultaneously activate the remodeling process. These two processes both require additional energy and the HF diet could have caused enough metabolic derangements to alter them. In previous studies, PO rodent models the HF diet was started at the time of surgery. This may explain why the authors did not observe any increased LV hypertrophy in their model despite similar metabolic assessments at the end of their protocol [7, 8].

The fatty acid composition of the HF diet may influence its effects on the heart. In our study, the content of saturated and monounsaturated fatty acids was in excess of 80% of total fat. A higher content in polyunsaturated fatty acids may have led to different results. It was shown that a diet rich in omega-3 polyunsaturated fatty acid and complex carbohydrates could confer a “lipo-protection” and decrease LV hypertrophy in an pressure overload mouse model [37, 38]. This observation suggests that the composition of a HF diet specifically influence the hypertrophic response of the overloaded heart. These alternate high-fat diets with a “better” fat profile will need to be tested in upcoming protocols.

In addition to perturbing myocardial FAO, we observed that glycolysis seemed altered by the HF diet. AR LVH is associated with a shift in energy preference towards glucose [15]. We showed that Glut4 translocation to the membrane is increased in AR animals suggesting an increased glucose uptake capacity. The HF diet completely obliterated this increase thus probably impairing the expected metabolic switch. This, in addition of probably less efficient FAO in the myocardium of AR rats fed with the HF diet could lead to increased lipotoxicity and poorer survival.

The overall oxidative capacity of the AR myocardium did not seem to be negatively altered by the HF diet. It has been reported that a HF diet in healthy rats was associated with increased FAO and oxygen consumption without an increase in function and thus with a decrease in cardiac efficiency. Uncoupling was also increased in the mitochondria, maybe as a protective mechanism against reactive oxygen species (ROS) production [39, 40]. Our results suggest a similar status in our SHF animals. In AR animals, uncoupling seems less active as suggested by the return to normal UCP3 expression. This could lessen the mitochondrial capacity to inhibit ROS production.

The exact reason why survival was decreased in the AR-HF group cannot be determined with certainty. Survival in humans with AR is directly related to LV dilatation and hypertrophy which is in line with our animal findings. Larger, more hypertrophied hearts are obviously more fragile and may be prone to pro-arrhythmia and sudden death. We did not observe any clear morphological changes in the hearts of sham-operated animals fed with the HF diet even though they were clearly overweight compared to animal on the normal diet. Recently, another study on Wistar rats has shown that a HF diet caused LV hypertrophy in healthy normal animals after 15 weeks. The investigators noticed an increase in bogy weight, circulatory insulin and leptin levels as well as an increase in myocardial collagen deposition in their animals [27]. They also observed an increase in the papillary muscle stiffness suggesting diastolic dysfunction. In a shorter study (12 weeks) using Sprague–Dawley rats, Carroll and collaborators did not report any cardiac abnormalities [25]. Longer studies may be necessary to reveal the true effects of a HF diet in those animals.

## Conclusions

In conclusion, a HF diet rich in saturated fatty acids is associated with increased eccentric hypertrophy and poorer survival in a model of LV volume overload from severe AR. This suggests that dietary habits and obesity may influence the evolution of volume overload cardiomyopathy and may also possibly have an impact on survival. This could be explained in part, by the activation of pro-hypertrophic signals controlled by the GSK3β and FoxOs and an improper metabolic adaptation of the myocardium. This hypothesis could be tested in humans and the impact of dietary counseling and weight management in patients with VO should be emphasized.

## Electronic supplementary material

Additional file 1: **Chronic high-fat diet-induced obesity decreased survival and increased hypertrophy of rats with experimental eccentric hypertrophy from chronic aortic regurgitation.** (PDF 203 KB)
