# Supplementary material for: Chronic high-fat diet-induced obesity decreased survival and increased hypertrophy of rats with experimental eccentric hypertrophy from chronic aortic regurgitation
Source: BMC Cardiovasc Disord. 2014 Sep 24;14:123. doi: 10.1186/1471-2261-14-123 (PMC4189197; doi:10.1186/1471-2261-14-123)
Supplement: Supplementary file 1 — Additional file 1: Chronic high-fat diet-induced obesity decreased survival and increased hypertrophy of rats with experimental eccentric hypertrophy from chronic aortic regurgitation. (PDF 203 KB) [file 12872_2014_773_MOESM1_ESM.pdf]

Chronic high-fat diet-induced obesity decreased survival and increased hypertrophy of rats with experimental eccentric hypertrophy from chronic aortic regurgitation.

Wahiba Dhahri, Marie-Claude Drolet, Elise Roussel, Jacques Couet* and Marie Arsenault.

Groupe de Recherche en Valvulopathies, Centre de Recherche, Institut universitaire de cardiologie et de pneumologie de Québec, Université Laval, Québec, Canada

Data supplement

Methods :

Evaluation of LV fibrosis

Sections from paraffin-embedded mid-LV portions were stained using Trichrome-Masson coloration. Three sub-endocardial sections/slide from all surviving animals were analyzed for the evaluation of the proportion of LV sub-endocardial fibrosis as the blue (fibrosis)/red (myocytes) ratio using a computerized image analysis system (Image-Pro Plus, Version 4.5, Media Cybernetics, Silver Springs, MD). The sub-endocardial sections were defined as the inner third of the LV wall facing the LV cavity. Peri-vascular fibrosis was evaluated separately from the myocardial fibrosis. The % of peri-vascular fibrosis was evaluated similarly (fibrosis = blue) and is expressed as the ratio of total area of blue staining minus area of the vessel lumen divided by the total fibrosis area plus the blood vessel area. Results are expressed compared to sham control group.

Results:

Levels of sub-endocardial fibrosis ware elevated in AR animals compared to controls but no differences were observed in animals fed the HF diet compared to the ones receiving control chow (Fig. S1). A similar pattern was observed in terms of peri-vascular fibrosis where AR animals had an increased area of fibrosis surrounding blood vessels but the HR diet did not impact this parameter (Fig. S2 A-B).

Sub-endocardial fibrosis (%)

Figure S1: Left ventricular fibrosis, extra-cellular matrix (ECM) remodeling and after 7 months in AR rats fed or not with HF chow. Quantification of sub-endocardial fibrosis by blue/red ratio from trichrome-Masson stained LV sections. Results are reported in as mean ± SEM (n=6). ***: $p<0.001$ vs.sham groups.

A

Peri-vascular fibrosis
(fold change relative to SC)

B

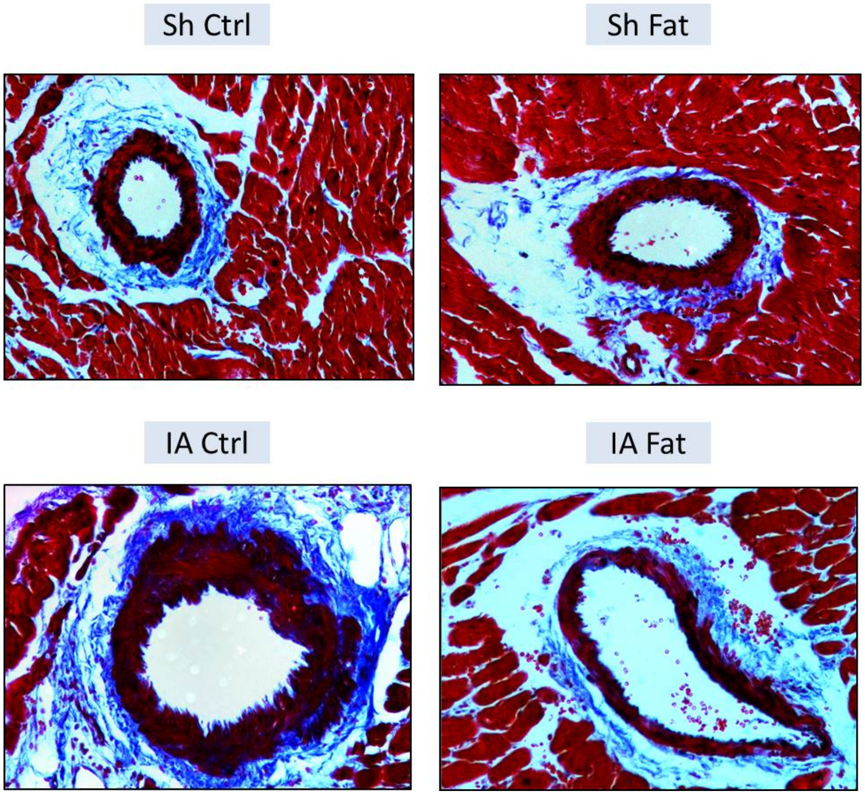

Figure S2: Myocardial peri-vascular fibrosis after 7 months in AR rats fed or not with HF chow. Quantification (A) of per-vascular fibrosis was performed as described in the Methods section. Results are reported in as mean ± SEM (n=6). *: $p<0.05$ vs.sham groups. Representative views are illustrated below (B).
